# Challenges and opportunities in cultivating medical students’ competencies: Participatory action research from a hierarchical cultural setting

**DOI:** 10.1080/10872981.2023.2185122

**Published:** 2023-03-03

**Authors:** Ardi Findyartini, Nur Afrainin Syah, Astrid Pratidina Susilo, Hikmawati Nurokhmanti, Nurul Qomariyah, Nadia Greviana, Dina Qurratu Ainin, Sylvia Mustika Sari, Mora Claramita

**Affiliations:** a Department of Medical Education, Faculty of Medicine, Universitas Indonesia, Jakarta, Indonesia; b Medical Education Center, Indonesia Medical Education and Research Institute, Faculty of Medicine, Universitas Indonesia, Jakarta, Indonesia; c Indonesian College of Health Professions Education – IAM-HPE, Yogyakarta, Indonesia; d Department of Medical Education, Faculty of Medicine, Universitas Andalas, Padang, Indonesia; e Department of Medical Education and Bioethics, Faculty of Medicine, Universitas Surabaya, Surabaya, Indonesia; f Department of Medical Education and Bioethics, Faculty of Medicine, Nursing and Health Sciences, Universitas Gadjah Mada, Yogyakarta, Indonesia; g Medical Education Unit, Faculty of Medicine, Universitas Ahmad Dahlan, Yogyakarta, Indonesia; h Medical Education Unit, Faculty of Medicine, Universitas Al Azhar, Mataram, West Nusa-Tenggara, Indonesia; i Medical Education Unit, Faculty of Medicine, Universitas Ahmad Yani, Bandung, Indonesia

**Keywords:** Student-centered learning, mentorship in medical education, student-teacher relationship, summative-formative assessment, constructive feedback, student-reflection

## Abstract

**Backgrounds:**

Research concerning student-centered learning (SCL) recommends a comprehensive assessment of medical students’ competencies including their personal and professional characters. Accordingly, nurturing future doctors should be in a continuous mentorship program. However, in a hierarchical culture, communication is one-way with limited feedback and reflection. We aimed to explore challenges and opportunities for SCL implementation in medical schools in this cultural setting necessary for a globally interdependent world.

**Methods:**

Two cycles of participatory action research (PAR) were conducted, involving medical students and teachers in Indonesia. A national conference on SCL principles was conducted between the cycles, also the SCL modules were developed for each institution and feedback was shared. Twelve focus group discussions were conducted (before and after the module development), with 37 medical teachers and 48 medical students from 7 faculties of medicine across Indonesia at various levels accreditation. Following verbatim transcriptions, a thematic analysis was conducted.

**Results and Discussions:**

In cycle 1 PAR, some challenges in implementing SCL were identified: lack of constructive feedback, overloaded content, summative-based assessment, hierarchical culture environment, and teachers’ dilemma of committed time between patient-care and education. In cycle 2, several opportunities to approach the SCL were proposed: a faculty development program on mentorship, students’ reflection guides and training, a more longitudinal assessment system, also a more supportive government policy on the human resources system.

**Conclusions:**

The main challenge of fostering student-centered learning revealed in this study was a teacher-centered learning tendency in the medical curriculum. The weighting towards summative assessment and the national educational policy drive the curriculum like a ‘domino effect’, away from the expected student-centered learning principles. However, using a participative method, students and teachers could identify opportunities and articulate their educational needs, i.e., a partnership-mentorship program, as a significant step toward student-centered learning in this cultural context.

## Backgrounds

The global values on student-centered learning (SCL) influence the development of medical competencies across wide-ranging areas. Harden (2018) clearly described the application of SCL in the medical curriculum as involving students as active partners in learning rather than passive clients [1]. Students also need to develop their learning goals with the teachers, and as a result, the evaluations move from an assessment of what has been learned (assessment of learning) to an assessment for further learning (assessment for learning). Accordingly, to apply the SCL principles toward life-long learning for future doctors, medical students need to master complex abilities. The theory of SCL is based on the socio-constructivist theory, in which learning occurs based on observation, imitation, and modelling; all of which are done through social interactions [2]. Therefore, medical students should not only master medical knowledge, but also develop complex abilities characterized by continuous interaction and mutual dialogue with their teachers, peers, and furthermore in the professional capacity, with their patients, as mentioned in a global competence-based medical education framework [3]. Complex skills such as critical thinking, social intelligence, novel and adaptive thinking, cross-cultural competency, communication, and teamwork can strengthen medical doctors’ personal and professional capacity in dealing with the challenges of interdependent work, as recommended in 21st-century learning skills [4–8]. Continuous mentorship, with periodic constructive feedback and reflection processes, is essential to facilitate the required abilities for the future workforce of medical doctors and health professionals [9–12].

Mentorship should be personalized to the individual students’ needs in terms of pace, duration, and learning approaches. The meaningful conversations between the two parties encourage critical and reflective thinking and learners’ growth. Such discussions should be bidirectional and conducted in a safe learning climate allowing the development of trust, a positive growth mind-set, and learning goal orientation. Teachers should be able to create a safe environment that is conducive to learning and take dynamic roles as role models, mentors, information providers, assessors, and facilitators [13,14].

It has been realized that personal and professional development should consider the socio-cultural factors involved in the curriculum implementation and teaching-learning process [9,10]. Studies show that learning and organizational cultures contribute to feedback-seeking behaviors where the teachers’ initiatives and positions as experts are critical in a hierarchical culture [9,10]. It is also found that unidirectional and top-down feedback is more prominent in the hierarchical culture, including in Indonesia as the setting of this study [15,16]. Hierarchical culture is characterized by a gap created by the social power structure due to people’s older age, knowledge, and positions. The hierarchical and cultural dimensions described in Hofstede’s study can be found in many regions, including Asia, predominantly Southeast Asian, South Asian, Mediterranean, African, and Latin American countries [17]. Considering the global movement of people in the present interdependent world, the challenges and opportunities found in this study can also be concerns in other regions.

Nurturing students’ personal and professional growth needs meaningful and continuous conversations between teachers and students and requires the pro-activeness of both parties. This supportive learning environment can be challenging in a hierarchical culture where mutual dialogue is rare and communication is a relatively one-way style [18]. However, the same study also showed that Indonesian society actually wants a more partnership relationship in doctor-patient communication [18]. The health professional practitioners in this study context also perceived that learning soft skills is essential in the medical curriculum as recommended by the 21^st^ century learning skills references [19]. As a result, participants in that study could endorse the role-play method instead of lecturing when teaching soft skills, although lecturing was still the central method in this context [19]. Other evidence from the same settings also found that mutual feedback is needed in workplace-based clinical settings, which is still rare [15].

Based on the literature above, it is important to discover challenges and opportunities to approach SCL in medical education (in a hierarchical culture, where dialogue and feedback are limited), by exploring medical students’ and teachers’ perceptions. In a hierarchical cultural context, problems and solutions should come from the people instead of a scholar since that may cause even more social distance and a lack of ownership of the issues and programs. Therefore, we used participatory action research (PAR) for the design of this study.

## Methods

### Context

#### The culture

Indonesia, the biggest archipelago and fourth most populated country in the world, is located in South East Asia [20] and is characterized by a hierarchical culture [15–18].

#### The medical curriculum in Indonesia

Medical education in this country consists of undergraduate medical education taking high school graduates, with a one-year internship program afterward. When the study was done in 2020, there were 90 faculties of medicine in Indonesia. There were 5 medical schools considered as the top rank among 20 with the highest level of accreditation, and about 30 with a medium level of certification, while the rest were emerging schools. These medical schools have high school graduate’s intake and are graduating medical doctors after 5.5–6 years of education (3.5–4 years of preclinical and 2 years of clinical studies) who can directly apply for professional practice. The Indonesian national standards of the medical curriculum have endorsed the competency-based approach with some attempts to increase SCL processes. All specializations are graduate programs, but Family Medicine has been just recently established in only a limited number of universities.

#### The professional practice

The basic medical doctors/MD, about 120 million registered to the Indonesian Medical Council, are 80% of the total doctors who do professional practices, whereas the graduate specialists/MD-Specialist are 20%. They all serve the 273.5 million Indonesians living on the five biggest and smaller islands [20,21]. Although the number of doctors seems sufficient for the total population, the unequal distribution of doctors has been a national problem for decades because nearly 80% practice on Java Island. Moreover, all specialists are allowed to practice in three different hospitals simultaneously, and the number of patients determines their salaries.

### Design

The design of this study is participatory action research or PAR [22–25], which is defined as ‘Communities of inquiry and action evolve and address questions and issues that are significant for those who participate as co-researchers’ [22]. The theory that underlines the PAR approach in medical education research involves co-creating and co-designing shared goals [24]. The authors in this study came from seven medical schools across the Indonesian archipelago with different levels of accreditation (the highest level, medium, and a new school), from Sumatera (the western part of the Indonesian archipelago), different places in Java (mid-southern part of Indonesia), and Lombok – West Nusa Tenggara (eastern part of Indonesia) islands who joined a national college of health professions education.

Accordingly, the design of this study was based on a series of discussions with these authors since the early pandemic situation in 2020 via online meetings. They contributed to the research questions of this study, methods, and later on results, analysis, discussions, and all other aspects of this paper. As representatives of their institutions or the demographic scope where they work, the authors were involved in the design of two cycles of PAR to invite more participation from students and staff members from their medical schools among others, to explore the problems and possible alternative solutions, for the research question. Furthermore, we submitted the proposal to get financial support from the Ministry of Education and Research Republic of Indonesia (MoER). The Medical and Health Research Ethics Committee approved the study at the Faculty of Medicine, Public Health and Nursing, Universitas Gadjah Mada, Yogyakarta, Indonesia with No. KE-FK-0598-EC-2020.

### Participants

Prior to the PAR, as we explained above, nine medical educationalists from seven medical schools in Indonesia were involved as researchers in this study. Then, the two cycles of PAR involved 37 medical teachers from preclinical and clinical stages of undergraduate medical programs and residency programs, 48 students from preclinical and clinical stages, and residents from specialty programs were invited to participate in this study. To capture variations of perspectives, we involved medical teachers and medical students from different medical schools in Indonesia, both from public and private universities. Invitation to participate was extended by the authors who joined the Indonesian College of Health Professions Education to the Heads of the Medical Education Unit (MEU), in the faculties of medicine from west Sumatera province, Java (the capital, west, middle, special region, and east provinces), West and East Nusa Tenggara provinces. The head of MEU then recommended its students and staff to participate in this study. We understand the non-randomized sampling created a limitation of the study due to the constraints of reaching out to the participants across several islands. All communication was conducted through electronic mail during the early pandemic situation.

Authors then contacted the potential participants through text messages, and upon their consent to join, we purposely grouped them with consideration of maximum variation sampling based on (1) gender, (2) preclinical/clinical stages of education/teaching, and (3) accreditation level of the medical schools of origin. The medical teachers consisted of 12 preclinical teachers, 12 clinical teachers, and 13 medical educators of preclinical or clinical teachers working in the medical education unit/department. The minimum working-year criteria for participation was 5-year. The student groups were comprised of 15 residents (all fields), 15 clerkships students, and 18 undergraduate students. The characteristics of the participants are illustrated in Table 1.

**Table 1.** Characteristics of the FGDs’ participants.

Participants		Gender		Department	Region
Teachers	Amount	Male	Female		
Clinical	12	4	8	Pediatric (2 female) Surgery (1 male) Eye (1 female) Pharmacologist (1 female) Anesthesiologist (1 male) Sports medicine (1 female) Internal medicine (2 males) Neurologist (2 females) OB (1 female)	2 Sumatera 1 Yogyakarta 2 West Java 7 Capital city
Pre-clinical	12	2	10	Pharmacology (1 female) Biochemistry (3 females, 1 male) Physiology (2 females) Midwifery (3 females) Anatomy (1 female) Public health (1 male)	5 Yogyakarta 3 West Java 4 West Sumatera
Medical educators	13	1	12	Medical Educ. Unit (1 male, 12 females)	2 West Sumatera 2 Capital city 4 West Java 5 Mid Java
Total	37	7	30		
Students					
Residents	15	6	9	Pediatric (2 females) DV (1 female, 1 male) OB (2 females, 2 males) ENT (2 female, 1 male) Internal Medicine (2 females, 2 males)	3 West Sumatera 6 Capital City 3 Mid Java 3 East Java
Clinical clerkship	15	6	9	All students finished final rotation	4 Sumatera (west Indonesia) 5 Capital City 3 Kalimantan/Borneo 3 East Java
Pre-clinical	18	4	14	Students finished their final semester before clerkships	6 Sumatera (west Indonesia) 6 Capital City 2 Yogyakarta 4 East Java
Total	48	16	32		

### Data collections

Led by the authors based on the agreed research questions and methods, a series of 12 focus group discussions (FGDs) for cycles one and two of PAR were completed. The FGDs consisted of participants with similar backgrounds to create a safer environment for sharing experiences, e.g., FGDs with teachers were separated from those with students, clinical teachers were separated from pre-clinical teachers, and also residents were separated from undergraduate students. All FGDs were conducted via video conference application, given the pandemic condition at the time of the study. We understood the inconvenience the participants might have felt since they did not know each other and should discuss these sensitive issues in an online meeting. Therefore, adequate information concerning the research goals and the consent form were delivered and obtained prior to each FGD, and participants could decline to participate at any time if they wanted.

**Figure 1.**
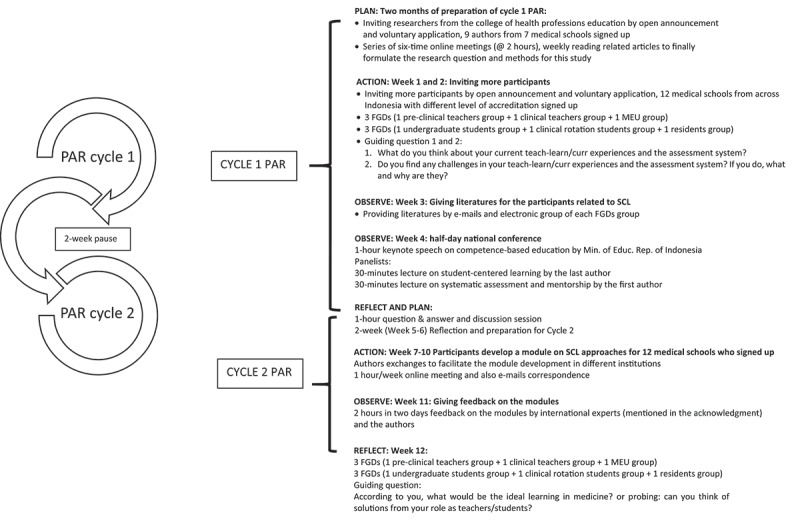
Cycles of the participatory action research in this study.

The detailed timeline and procedures of this study are illustrated in Figure 1, following four stages of each cycle of PAR: Planning, Action, Observation, and Reflection. Two cycles of the PAR described as follows, Cycle 1: recruitment of other participants from more medical schools interested in developing SCL modules for their institutions. Twelve medical schools signed up. We did the FGDs to explore challenges in SCL implementation in three FGDs consisting of 3 groups of different teachers (pre-clinical, clinical, and MEU) and 3 other FGDs with groups of students [undergraduate, clerkship, and residents). We also conducted a national conference on SCL and a formative assessment, supported by the MoER. This national conference was also open for participants outside this study due to the importance of distributing the information of the SCL approach. In Cycle 2, the 12 medical schools were asked to develop an SCL module that provides guidance tailored to each of their institutions toward the SCL learning approach. The authors offered weekly advice for each school’s team. Two well-known international experts in medical education (mentioned in the acknowledgments) provided feedback on each module. Finally, the last half of the sessions of the 6 FGDs were conducted for reflection and evaluation purposes.

### Instruments

The authors as moderators of the FGDs used these questions to guide the discussions:

Questions on the first cycle of the PAR for all groups:

1. What do you think about your current teaching-learning/curriculum experiences and the assessment system?
2. Are there any challenges in your teaching-learning/curriculum and the assessment system? If you do, what and why are they?

A question on the second PAR cycle:

According to you, what would be the ideal learning in medicine? Or probing: can you think of solutions from your role as teachers/students?

These questions were more general, and we did not guide the participants into discussing specific themes, i.e., culture. We explored participants’ perspectives regarding the questions as deeply as possible.

### Data analysis

All FGDs were transcribed verbatim. Steps in the thematic analysis following Braun and Clarke’s steps [25]: 1. Familiarizing the data. All authors initiated the thematic analysis by engaging with the data by reading the transcripts of minimum three times, 2. Generating initial codes. The authors revealed relevant categories of the transcripts, which were all done manually based on the experiences in qualitative studies of the authors, 3. Searching for themes. All authors then met regularly for a 6-week iterative process to discuss the themes list, 4. Reviewing themes. Any disagreements were discussed among the authors, 5. Producing the themes. All authors complete the subsequent analysis and decide on the final names/words for each theme agreed upon until no new themes emerged or data were saturated, and 6. Reporting. All authors discussed the data and reported the final themes.

## Results

**Figure 2.**
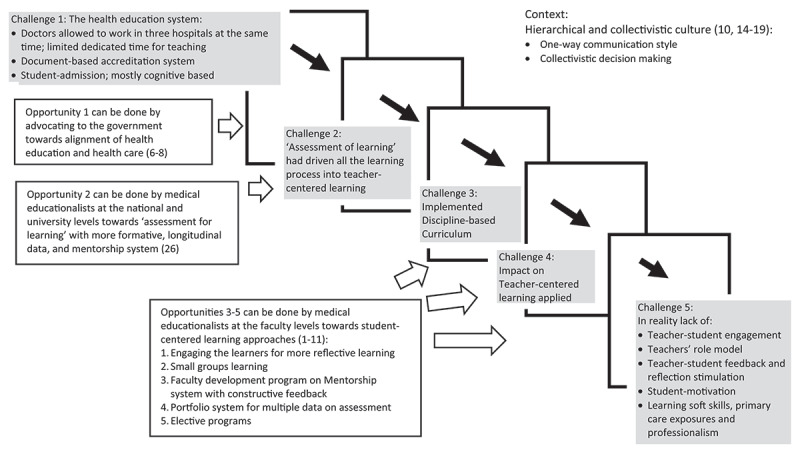
A ‘domino-card’ effect of the learning process in medical education taken form the focus group discussions of the cycles 1 and 2 of the PAR in this study, revealing challenges and opportunities towards student-centered learning approach.

This study explores teachers’ and students’ perceptions regarding challenges in SCL implementation in medical education and possible approaches. We found a ‘domino-card’ effect that is illustrated by the first factor that influences the other elements to fail or to be unsuccessful. The ultimate result was the opposite of SCL (more on teacher-centered learning/TCL) in the medical education settings that we studied (as described in Figure 2). The first factor starts with the ‘health education system’ that allows doctors to work in three clinical settings simultaneously, which leads to potential dilemmas encountered by medical teachers in providing patient-care services or training the students. Conflicting commitments in using their time can cause less time to educate the students. In this case, the tendency for more teacher-centered learning is noticeable. The teaching process also has limited time for observations. Therefore, there is little feedback for the students.

Included in the ‘health education system’ is also a factor of the national accreditation system, which mainly uses document-based checklists instead of a more self-evaluation or strength-based approach. At this point, the quality of the institutions’ and students’ personal and professional development can be regarded as a minor component of the accreditation system, compared to the quantity of the document. Additionally, as part of this prime factor, the student-admission system still mainly uses cognitive-based tests with a minimum exploration of students’ interests and motivation to choose medical schools. Therefore, individual self-reflection and learning attitude are also already trivial fragments since the beginning of medical education, compared to the score of their admission tests.

The second factor illustrated in our ‘domino chain’ is the ‘national exit examination at the end of medical education. The impact of the national exit exam is enormous. The medical education process may even be farther away from student-centered learning because students’ learning seems only to be directed toward the final results of medical education in the teachers’ perceptions: to pass a high stake examination. The exam results are considered for medical schools to obtain a better accreditation level, rather than providing feedback for learning for students and institutions.

These series of ‘top-down’ rules and regulations force the implementation of medical education into the third factor of our ‘domino-cards,’ which is a ‘discipline-based curriculum’ rather than an expected integrative one. Dialogue between teachers during the curriculum development tends to be severely restricted due to limited time and commitment for teaching and the need to devote more time to preparing the documents for accreditation, the national exam, and professional practices. Therefore, teachers find it is more effective to only follow the chosen topics for lectures than designing a more integrated teaching approach. Consequently, this element of ‘teacher-centered learning style’ disregards the process of nurturing and mentoring medical students throughout the curriculum. Ultimately, the impacts of the current medical education found in this study include a lack of teacher-student engagement, and a lack of mutual dialogue, which possibly contribute to decreasing student motivation for self-directed learning.

On top of these problems, the context of the culture accepts the wide power distance between the perceived higher-lower social hierarchies or the teacher-student relationship. In Indonesia, society approves the above rules and regulations to so-called ‘standardize’ all medical doctors. Possibly, the need for students’ personal and professional development is neglected. As a result, the complex skills recommended to be mastered by medical doctors in the student-centered learning principles seem challenging to accomplish. Figure 2 describes the relationships of the themes using the domino card-effect analogy.

To have alternative solutions regarding these problems bound within the hierarchical cultural background of this study, we benefited from the PAR by providing continuous dialogue between researchers and participants, so issues and solutions were derived from the society. The medical students and teachers in this study were engaged with a sense of more ownership rather than if we did a formal training or an experimental study on SCL. Figure 2 shows some of the possible solutions proposed to advocate the SCL approach, which medical educationalists and medical teachers at the national and university levels can do.

The thematic analysis of challenges in implementing SCL medical education obtained from the FGDs before the national conference and module development is presented in Table 2. Whereas Table 3 indicates the opportunities for further learning for faculty and students to approach the SCL obtained from the last stage of the second PAR cycle.

**Table 2.** Themes and categories derived from participants’ answers for questions 1 and 2 of the FGDs.

		Overall frequency (ranked from the most frequent spoken concern)		
Final themes	Categories	Total	Articulated by lecturer (6 groups N = 37)	Articulated by students (6 groups N = 48)
Theme 1: Teacher centered learning tendency	Lack of conception of facilitating learning	48	45	3
	Teacher-centered learning	40	29	11
	Lack of constructive feedback	36	10	26
Theme 2: Neglected nurturing on students’ personal and professional development	Neglected individual uniqueness	31	22	9
	Neglected training on Soft Skills	31	26	5
Theme 3: The top-down assessment and overall educational system	Summative assessment had driven curriculum into current situation	31	28	3
Theme 4: The hierarchical culture influence	FOG (Fear-Obligation-Guilty) culture	22	18	4
	Hierarchical culture (the context)	18	11	7
Theme 5: The impact on the teachers’ attitude based on above themes	Lack of proper role model	14	5	9

**Table 3.** Themes and categories derived from participants’ answers for question 3 of the FGDs.

			Overall frequency (ranked from the most frequent spoken concern)		
Final themes		Categories	Total	Articulated by lecturer (6 groups N = 37)	Articulated by students (6 groups N = 48)
	Theme 1: Faculty development on continuous learning, mentorship, and assessment*	Faculty development series on constructive feedback*)	36	36	
Theme 2: Supportive educational and healthcare system		Commitment from the Min. of Educ. on the policy regarding: Scheduled appointments for patients and students More formative accreditation system Proper Remuneration	31	6	1
	Theme 1: Faculty development on continuous learning, mentorship, and assessment*	More formative assessment*) Individualized feedback Portfolio	31	15	16
Theme 3: The need for reflection-learning training and guide**		Student development guidance and training series**)	24		24
	Theme 1: Faculty development on continuous learning, mentorship, and assessment*	The need for constructive feedback*)	25	9	16
		The need for good role models as medical teachers. This can be done if the educational policy supports *)	21	11	10
		Appraise individual uniqueness	15	12	3
		The need for professional training and guidance*)	12	10	2
		Challenges of hierarchical and collectivist culture within the generation gap *)	10	5	5
Theme 3: The need for reflection-learning training and guide**		The need for reflection-learning training and guidance**)	8	2	6
		Equal opportunity to learn soft and hard skills**)	7	4	3
		More experiential learning**)	6	3	3

Some of the quotations concerning each of the themes found as ‘SCL challenges’ in this study are presented in Table 1. The quotations were selected from the first sessions of the FGDs to answer the questions 1 and 2 in the FGDs’ guide.

## The challenges to implement a student-centered learning approach in this study setting

### Theme 1: teacher-centered learning tendency

Our study underscores some issues regarding various conceptions of facilitating learning, teacher-centered learning, and the lack of consideration of individual uniqueness, lack of role modelling and lack of constructive feedback. The various conceptions of learning are described below:

“What is the role of a mentor? They just sign our subject credit-semester and nothing else.” (FGD with undergraduate students)

“We should write our reflection on the portfolio, but it is so rare that we get the feedback. Our writings just there on the desk.” (FGD with the residents)

“We tend to speed up when we teach our students; ask them to be self-directed learners, encourage them to read a lot of textbooks. We have to guide them to ‘digest’ the abundant information well.” (FGD with preclinical year teachers)

“I am worried that the students would not be able to understand this unless I teach them’” (FGD with pre-clinical teachers)

“There might be some feedback, yet they are not constructive. A lot of teachers haven’t understood how to provide constructive feedback” (FGD with MEU teachers)

### Theme 2: the neglected process of nurturing students’ personal and professional development

There were discussions and recognition that soft-skills such as professional behavior, communication, self-awareness and self-reflection are necessary. However, their coverage and assessment, especially in practice in the current curriculum, were considered minimum. Some of the critiques from our FGDs’ participants are as follows:

“We [students] need to be encouraged to internalize and practice [the professional behaviors]. It is beyond cognitive and skills [aspects].” (FGD with clinical year students)

“They teach us with a module of ‘Being emphatic doctors’, but all was lectures in it and none was touching on to give feedback to our behavior. ‘Breaking bad news’ is like a never be a reality skill.” (FGD with the residents)

“We have cognitive and skills as heavy in the curriculum. Students’ attitudes and behaviors [development] are not well covered” (FGD with preclinical year teachers)

“[I find] that assessing professional behaviors is very challenging. We do know the theories. Yet, the practice is not that easy.” (FGD with clinical teachers)

“Not all of us understand that soft skills development requires [longitudinal] and continuous process starting from the students commence their medical education.”(FGD with MEU teachers)

### Theme 3: the top-down assessment and overall educational system

The current curriculum emphasizes summative rather than formative assessment. Discussions also highlighted the strong practices of hierarchical culture and content/discipline-based approach. The teachers and students identified the findings consistently. First, the reliance on summative assessment is strongly embedded in the curriculum given the current higher education assessment system, hence ‘students do what we inspect, rather than what we expect’. Despite the effort to implement formative assessments, the use of the current scoring system was problematic. For example, some FGD participants shared the following:

“Why are students still very score-oriented? Because I think that what has been emphasized by our medical school: You need to pass this course with minimum score of 65 for example. And there is a consistent endorsement of high GPA in our current stage so that we can continue to future postgraduate programs.” (FGD with undergraduate students)

“Time allocation for lectures are away above time for providing feedback and mentorship.” (FGD with pre-clinical teachers)

“There is a gap between pre and clinical curriculum. The knowledge seems fading away when the students move into clinical years” (FGDs with pre-clinical teachers and clinical teachers)

“We are enforced by the current university system to use scoring classification: A, A+, B, C, and so on, when actually the scoring cannot always capture the professional [development] goals of our medical student … ” (FGD with clinical teachers)

“As the coordinator of the residential program, I practiced the 360 degree feedback to our residents, but finally we got all good scores for each one, without any feedback in it. I think they ‘cooperate’ with each other about the scores” (FGD with clinical teachers)

“When we try to use Mini-CEX form, [our teachers] find it easier to just put numbers in the columns, when what we expect is [narrative] feedback.” (FGD with MEU teachers)

### Theme 4: the hierarchical culture influence during curriculum implementation

Our study also identified challenges which come from conflicting learning experiences, the wide power-distance culture, and the various students’ behaviors and attitudes concerning learning. For example, the responses included the following:

*“We need to study from the expert. Otherwise, if only among peer-students during tutorial, we do not get the correct information.” (FGD with undergraduate students)*

“We got scolded, or sometimes humiliated. We [expect] to obtain constructive feedback. Sometimes, our teachers seem to have very high expectations of us, and get disappointed when we cannot respond or perform well. They maybe forget that we are still novices” (FGD with clinical year students)

“The gap of senior-junior relationships is always there. Consequently, we [the juniors] should always initiate a well-mannered behavior and communication with our seniors … ” (FGD with the resident)

“We have feedback provision training in place, the teachers are willing to provide feedback, and the students are also eager to seek feedback. I still find somehow that the practice is not consistent. [The feedback dialogue] It is not yet part of our culture”(FGD with clinical teacher)

“As a consultant, I have been studying and working for more than 30 years, I find myself a rather less self-confidence, I only got appreciation from the students, staffs, and nurses, but never from my seniors because they do not want me to be arrogant. Now, I realize that I should appreciate my students as part of their personal and professional development. I realize that I only have few knowledge, but I can help them [students] to organize it into the right folders (FGD with clinical teachers)

“I find that students are still ‘frightened’ to ask feedback from their teachers.“(FGD with MEU teachers)

### Theme 5: the impact on the teachers’ attitude

The context of the health care and educational system is already explained in the methodology section. Because one clinical MD can work in three different hospitals and the fact that current general practitioners only graduate from basic medical education without further postgraduate specialist training, variations of teachers’ attitudes can be found when dealing with students, for example:

“We have some friendly and engaging teachers. We also have teachers who do not listen well to us. Sometimes our teachers seem to ignore the age and knowledge gap, hence they do not adapt to the need to engage us well in the teaching sessions. Some other teachers were aware about this, and even use the technology well” (FGD with year undergraduate students)

“We face a lot of rescheduling in our clinical rotation when it comes to discussions with our supervisors. I however met this clinical teacher who give an excellent example on commitment to the schedule. He said to me,’ if you are expected to come at 7 am, it means you have to come 30 minutes before. There will be conditions when you have to reprioritize.’ I had a long discussion with him on the importance of professionalism in medicine, by providing a true example. I find this a very rare [opportunity].” (FGD with clinical year students)

“We send our students to different teaching hospitals where they meet rather glamorous specialist doctors. These are the role models the students encountered. When the graduates are needed to work in the rural areas with limited resources, they just find out a different reality.” (FGD with preclinical year teachers)

“The education and health care system should be organized better, i.e. the proper flat salary and maximum working-setting so that the doctors do not have to go here and there.” (FGD with clinical year teachers)

Table 2 presents some of the quotations concerning each of the themes of the opportunities to approach the SCL in medical education found in this study. The quotations were analyzed from the first sessions of FGDs to answer question 3 in the FGDs’ guide.

## The opportunities to approach student-centered learning in the setting of this study

### Theme 1: the need for series of faculty development on continuous learning, mentorship, and assessment

We identified the shared insights that underscored the students’ and the teachers’ need for sustained and continuous learning and assessment approaches such as mentorship programs and formative assessment. Those approaches are expected to facilitate more dialogic and specific feedback, as expressed in the following PAR participants’ responses.

We would like to be heard, and to have dialogue with the teachers. (FGD with undergraduate students)

‘Feedback on individual basis is highly needed.’ (FGD with clinical year students)

The mentor for medical students should be well and continuously trained with the guidance of a proper mentorship program. (FGD with pre-clinical teachers)

The spirit of assessment is during the mentoring development and the formative one. Summative; [on the other hand] can be just a fortune. (FGD with clinical teachers)

### Theme 2: the need for more supportive educational and health care system

As one of the recognized opportunities in the SCL implementation in the study context, we found participants’ awareness of the need for better support in institutional or national policy for health professions education and health care. This opportunity was described by several participants, for example:

Time allocation for adequate feedback is important, in regards to the overall human resources system. Supervisors need a strong support-system to divide their time for patient care, research, and students. (FGD with clinical teachers)

Continuous process of learning should be systematically recorded. (FGD with clinical teachers)

Value is important, progress over the results is also very important, but I do not know how to put it into a system. The full-time equivalent? (FGD with MEU teachers)

### Theme 3: the need for reflection-learning training and guidance for medical students

From the students’ point of view, we found the awareness of the reflective learning approach facilitates by supportive feedback from teachers and coach-mentoring activities in self-reflection skills, in which teachers could engage and guide students’ professional development.

You said we should be an independent learner, so we do not need any feedback? [Referring to the other student]. No, I think as an independent learner we shall always learn from any feedback to make us a better person. (FGD with undergraduate students)

‘We need guidance and training to stimulate reflection.’ (FGD with clinical year students)

How much do you want to know and understand the patients? Should be continuously nurtured especially for the residents. (FGD with clinical year students)

We need to prepare the generation z who already familiar with digital information literacy, and the generation gap with the teachers. (FGD with MEU teachers)

## Discussions

Our study highlights that cultivating medical students’ complex abilities in the current study setting should consider cultural challenges since they may prevent mutual interactions and meaningful dialogue between teachers and students during the education years. The study strengthens previous study findings in a similar setting where teachers’ positions are considered superior in their position of authority and bidirectional constructive feedback is still rare or inconsistent. We attempted to discuss our study findings using the domino-card model to deliberately explain the relationships of the themes and subthemes and the domino effects of the system, teacher, and student factors.

The challenges identified in this study for the accomplishments of competent medical doctors (that include both medical knowledge and skills, personal and professional characters) seemed to be strongly influenced by the culture that accepts more instructions or rules and regulations from the ‘above’ perceived authorities. First, despite aspirations from the teachers and students regarding the need for constructive feedback and a more formative assessment, the accreditation system and assessment policy at the national and university levels emphasize the summative results and numeric evaluation of institutions and students’ performance. Changing the culture towards a more systematic and longitudinal assessment, i.e., programmatic assessment (where continuous dialogue enforcing feedback and reflection is the key for further learning) is complex and requires an understanding of the common beliefs held by the parties involved in the education system [26]. Therefore, the current challenges might hinder the effort of the SCL implementation if they are not mitigated and managed well.

Second, hierarchical culture was also seen in the tendency toward the discipline/content-based approach and teacher-centeredness. In this regard, teachers were placed as decision-makers of their content expertise that can be delivered in the curriculum. The focus was more on assuring students cover the content rather than challenging them to think critically and linking to their previous knowledge. In addition, the representativeness of the content in the curriculum was considered critical in this setting. Beyond each teacher’s content expertise in attempting to increase the relevance of the content with clinical or practical applications and dealing with students’ variations, providing additional SCL opportunities was challenging for the teachers. Such findings are consistent with studies conducted in a similar setting [14–16]. In addition to the social power distance between teachers and students, it was notable that both teachers and students described the inconsistent practice of facilitative learning and dialogic feedback.

Third, teachers and students in this study were aware of the importance of professionalism and other soft skills’ development. While there had been courses and integrated approaches within the curriculum dedicated to this, they agreed that the implementation was not yet consistent, especially during the clinical years. It could be that the professionalism and relevant soft skills’ definitions were deemed unclear, especially at the practice level. Indeed, professionalism and its attributes can be defined differently in various settings [27,28]. The findings of this study suggest that the curriculum addressed the development of professionalism and soft skills mainly at the cognitive level. In addition, role modelling examples as the backbone of teaching professionalism and soft skills in practice [29] were exemplified by teachers in the preclinical and clinical years in this study.

However, students still felt that good role modelling was still rare. The role modelling by teachers in this setting would be central for teaching professionalism and soft skills given the hierarchical culture; that is, teachers practicing good examples of professionalism would strongly influence students’ attitudes and behavior. Nevertheless, the dialogues were probably still lacking between teachers and students to reflect on and internalize the good standards. Also, it was found that the assessment of professionalism implementation in practice was challenging due to reliance on the quantitative assessment. The room for improvement in this instance is large since professionalism can be assessed by different methods such as observed clinical encounters, self-assessment, multisource feedback, and simulations [30,31].

Fourth, students reported the fear-obligation-guilty (FOG) phenomena and variations in students’ behavior toward learning. A similar finding was also described by a study in Taiwan, another Asian country where medical students did not dare to give opinions because of their fear of negative outcomes [32]. The teachers in the current study were also aware that constructive feedback was not yet part of the culture due to several reasons, such as limited time to observe students’ performance and to discuss feedback and limited skills in providing feedback. From the students’ point of view, they understand the importance of feedback, yet they tend to be afraid to ask for feedback because they are anxious that they would be humiliated and feel very guilty afterward. Shame and guilt could affect students’ motivation, self-worth, and professional identity development [33]. The FOG phenomena may take place differently in different settings. Yet, it could be more influential towards students in the current setting since they might have felt discouraged to speak up and instead prioritize their acceptance and connectedness in the learning environment [34]. It is necessary to provide psychological safety, encouragement for students to seek feedback, and clear expectations from the teachers in feedback dialogue and follow-up. Considering the role of teachers in the current hierarchical setting, we suggest that the teachers can initiate a positive learning environment and create feedback conversations. Therefore, aligning the formative assessment system and building the capacity of the teachers to provide constructive feedback are very much needed in the current setting.

Medical curricula in the current setting should also explicitly and progressively develop professional attributes and soft skills. The development of professional character should be done systematically and longitudinally since the early preclinical period until the final clinical stages. Developing medical students’ competencies, including personal and professional characters, should also be centralized in a teacher-student interactive dialogue. On the one hand, teachers’ conception of teaching and their awareness to become positive role models and take the initiative in the feedback dialogue are critical. Faculty development aimed at increasing teachers’ skills to provide constructive feedback and encourage students’ reflections should be part of the systematic strategy. On the other hand, students should be encouraged to reflect on their performance, think more critically, engage better in their learning and seek feedback regularly. In the current setting, such encouragement requires the development of a positive learning environment.

We are aware of the study’s limitations: (1) The study was conducted in one country depicting a specific cultural context in relation to the efforts of nurturing medical students’ competencies. Therefore, it is best to interpret the study findings by considering the cultural contexts. We involved teachers and students from different medical schools in Indonesia in capturing various rich experiences in the teaching and learning of complex abilities. (2) The moderators of student FGDs in this study were medical teachers and researchers who might reflect some social power distance with the student respondents. We attempted to allocate moderators from different medical schools to the respondents and assured them that the data were confidential with no consequences for their assessment. Hence, we expected that their shared experiences were trustworthy. (3) The national conference and the SCL module development in between the two PAR cycles in this study can be analogous to an ‘intervention’, in which we realized where the gap of ‘competence’ between researchers and participants lies and we tried to get closer to increase their ‘motivation’ by offering continuous facilitation sessions [22–24].

Overall, the co-creation and co-design approaches as the methods of this study have successfully engaged students and teachers in revealing the problems and purposing plans for better medical education in our setting. The feedback conversations from the PAR cycles in this study have successfully invited ideas from the participants with a more partnership relationship and not only as users of the medical curriculum. This particular participatory research method is suitable for any cultural setting. However, specific advantages of the co-creation and co-design approaches for the hierarchical cultural backgrounds, such as in this study’s setting, include narrowing the social gaps between the participant-researcher, and collecting common goals from the participants from different across the country, where they might feel safer rather than if they propose their ideas or plans individually.

## Conclusions

The main challenge of approaching student-centered learning in medical education in this study was the applied deep-rooted judgmental scoring methods for individual assessments of future doctors and the institutional accreditation system, rather than acknowledging more narrative assessment strategy by emphasizing appreciative feedback and reflective learning approaches. These long-standing summative courses of action can be a part of accepting the social gaps of power, particularly in hierarchical cultural backgrounds that distinguish people’s positions in society. Therefore, the medical curriculum in this study was driven away from the expected SCL principles and tended to teacher-centered learning.

Using participative action research, students, teachers, and authors in this study can recognize opportunities for the student-centered learning approach, which follows global recommendations for 21st-century learning skills and educational principles for future doctors. The need for faculty development training for nurturing future doctors; by continuously having dialogue and providing guides on constructive and reflective learning, also the government support for the alignment between national education and health care services policy, was strongly expressed in this study. We hope this study will simulate significant changes towards better medical education and health care services in the study setting, regional, and global community.
